# Heat stress modulates the disruptive effects of *Eimeria maxima* infection on the ileum nutrient digestibility, molecular transporters, and tissue morphology in meat-type chickens

**DOI:** 10.1371/journal.pone.0269131

**Published:** 2022-06-03

**Authors:** Ahmed F. A. Ghareeb, Gustavo H. Schneiders, Jennifer N. Richter, James C. Foutz, Marie C. Milfort, Albert L. Fuller, Jianmin Yuan, Romdhane Rekaya, Samuel E. Aggrey

**Affiliations:** 1 Department of Poultry Science, University of Georgia, Athens, Georgia, United States of America; 2 State Key Laboratory of Animal Nutrition, College of Animal Science and Technology, China Agricultural University, Beijing, Peoples Republic of China; 3 Department of Animal and Dairy Science, University of Georgia, Athens, Georgia, United States of America; USDA-Agricultural Research Service, UNITED STATES

## Abstract

*Eimeria (E*.*) maxima* is one of the most pathogenic *Eimeria* spp persistently invading the middle jejunum and ileum, damaging the intestinal mucosa of chickens. Heat stress (HS) is a common stressor and equally contributes to inflammation and oxidative stress. We investigated the effect of *E*. *maxima* infection and HS on ileal digestibility, mRNA expression of nutrient transporters, and ileal tissue morphology in broiler chickens. There were four treatment groups: thermoneutral control (TNc), thermoneutral infected (TNi), heat stress control (HSc), and heat stress infected (HSi), 6 replicates each of 10 birds per treatment. Chickens were fed a diet containing 0.2% TiO_2_. At 6-day-post infection, ileal content and tissue were collected to quantify ileal digestibility of crude protein and fat, mRNA levels of nutrient transporters and histopathology. Growth and feed intake were reduced in all treatment groups, compared with the TNc. Contrary to expectation, the combination of two major stressors (*E*. *maxima* and HS) in the TNi group exhibited almost normal digestibility while only the TNi birds expressed severe digestibility depression, compared with the TNc group. The TNi group showed the lowest mRNA expression of the transporters: SGLT1, GLUT2-5-8-10-12, FABP1-2-6, and PEPT1 compared with the other treatment groups. The expression of the absorptive enterocytes’ gene markers (ACSL5, IAP, and SGLT1) supported by the ileal tissue morphology indicated that the TNi group had the highest enterocytic destruction. The expression of oxidative genes (iNOS and CYBB) dramatically increased only in the TNi group compared with the other treatment groups. Our results showed that exposing broiler chickens to HS can mitigate the disruptive effect of *E*. *maxima* on the ileal digestibility and absorption by limiting the parasite-induced tissue injury and suppressing the enterocytic inducible oxidative damage.

## Introduction

Coccidiosis is a parasitic infection caused by *Eimeria spp* and persistently affecting broiler chicken, causing high morbidity, impaired growth, and mortality [1]. Unlike many other protozoan parasites, *Eimeria spp* exclusively invades the intestinal epithelium showing high tissue specificity [2]. *Eimeria* (*E*.) *maxima* is characterized by producing the largest oocysts and male gamonts among the other species infecting chicken [3]. *E*. *maxima* particularly infects chickens at proximal and distal to Meckel’s diverticulum of the mid-gut and along the ileum and generates a huge number of merogonic stages, destroying the intestinal mucosa and excessively impacting the digestibility and absorption of most nutrients. Eventually, the intestine undergoes vast areas of enterocytic damage and epithelial sloughing, predisposing for the invasion of other pathogenic organisms [4, 5]. The shortening of the intestinal villi and crypt cell hyperplasia are remarkable consequences of *Eimeria* infection and reflect the pathological impact of the parasite on the intestinal tissue [6]. The severity of *E*. *maxima* infection depends on many factors, such as the strain, number of ingested sporulated oocytes, birds’ immunity status, and intestinal integrity [7, 8].

High ambient temperature is a widespread seasonal environmental stressor in broilers’ houses in temperate regions, and endemic in subtropical and tropical regions. Several studies have shown that exposing the broiler chickens to acute or chronic heat stress (HS) results in a severe reduction in feed intake and growth, and an increase of feed conversion ratio [9–11]. Even though HS slightly improves nutrient and amino acid digestibility, some reports have shown that such improvements are not statistically significant [10, 11].

The ileum plays a fundamental role in nutrient absorption by expressing various types of transporters and binding proteins. Monosaccharides are transported by a group of solute carriers and protein co-transporters that may or may not depend on insulin to be expressed on the enterocytic cell membrane [12]. Facultative glucose transporters (GLUTs) are responsible for transporting monosaccharide molecules in enterocytes and are classified based on phylogenetic structure into three classes: Class I includes GLUTs 1–4, and GLUT14 in human, class II is made up of GLUTs 5, 7, 9, and 11, class III consists of GLUTs 6, 8, 10, 12, and H^+^/myoinositol transporter (HMIT/GLUT13) [13, 14]. Enterocytes also express an active sodium-glucose symporter (SGLT1) on the apical membrane that utilizes the concentration gradient of Na^+^ to transfer glucose against its concentration [15]. Absorption of lipids and fatty acids occur through fatty acid-binding proteins (FABPs) and fatty acid transport proteins (FATPs) that are expressed on the apical membrane of the intestinal mucosal cells [16, 17]. The di- and tripeptides are absorbed via H^+^-dependent peptide transporters (PEPT1 and PEPT2), and peptide/histidine transporter 1 (PHT1) [18, 19]. The intestinal digestibility and mRNA expression of nutrient transporters have been used as a physiological and functional indicator of the enteric mucosal integrity and gut health of the broiler chickens under various pathological or stress conditions [20–22].

The acyl-CoA synthetase long-chain family member 5 (ACSL5) encodes for an enzyme responsible for activating the long-chain fatty acid to acyl-CoA that is required in various cellular functions [23]. Intestinal alkaline phosphatase (IAP) is a tissue-specific catalase enzyme that mediates the removal of phosphate groups from different cell molecules [23, 24]. The ACSL5, IAP, and SGLT1 (SLC5A1) genes are found to be highly expressed in the intestinal epithelia cells, especially the villus enterocytes, compared to the other intestinal cell types [24, 25]. The expression level of those genes, ACSL5, IAP, and SGLT1, can be used as a relative quantitative estimator for the abundance of villus absorptive enterocytes [26].

Our objective was to investigate the digestibility, molecular expression of nutrient transporters, ileum function and enterocyte damage in broiler chickens infected with *E*. *maxima* and reared under either thermoneutral or chronic HS condition. We hypothesized that the combined effects of HS and *E*. *maxima* infection would more gravely affect intestinal digestibility, function, and tissue architecture than their independent effects.

## Materials and methods

All experiments in this study were implemented under the Animal Use Proposal (AUP) number A2015 04–005 approved by the Animal Care and Use Committee (IACUC) of the University of Georgia.

### Experimental design and sampling

We conducted an experiment with two continues temperature levels (20°C and 35°C) and two infection levels (infected and control). *E*. *maxima* sporulated oocysts were obtained from a single oocyst cloning using North Carolina field strain. The infectious dose and dose preparation were detailed in Schneiders *et al*. [27]. One hundred and twenty 14-days old Ross708 broiler male chickens were randomly distributed into 12 cages and gavage-infected with 2x10^5^ *E*. *maxima* sporulated oocysts/bird suspended in distilled water and housed at two rooms of different temperature levels 20°C (TNi) or 35°C (HSi). Another 120 chickens were mock-infected with distilled water and randomly allocated into 12 cages in two other rooms at 20°C (TNc) or 35°C. The chickens were raised in their respective temperature continuously for 14 dpi. All chickens were raised in wired-floor cages, provided with *ad libitum* water and fed on a grower corn-soybean meal-based diet containing 0.2% titanium oxide. The experiment duration was two weeks. The chickens’ health and behavior were monitored twice daily. The human end point was considered to euthanize the bird upon showing inability to reach feed and water, sever respiratory distress, or severely emaciation (body score 1). The *E*. *maxima* infection was confirmed by detecting the oocysts shed in feces at 6 dpi and also by visualizing the coccidia stages in the histological images. The analysis of the feed is presented in S1 Table. Body weights were individually recorded on the day of infection (day 0) and at 4, 5, 6, 7, and 14 day-post-infection (dpi). Feed intake was recorded on 0, 7, and 14 dpi. At 6 dpi, seven chickens from each treatment group were randomly collected, and euthanized humanely by cervical dislocation to sample about 1 cm of whole ileum tissue (posterior to the Meckel’s diverticulum) (n = 5/treatment group). Ileum tissue samples were flushed with phosphate buffered saline, collected in cryovials and snap-frozen in liquid nitrogen and stored in -86°C for long-term storage (n = 5). Also, ileal contents were gently squeezed and collected in aluminum foil containers from all cages (n = 6/treatment). The cloacal temperature was recorded at 0, 3, and 6 dpi using CVS^®^ Health Digital Flexible Tip Thermometer (CVS Health, USA). Two mortalities were recorded in the HSi group, one found dead at 3 dpi and one humanly euthanized at 6 dpi.

### Apparent digestibility

Ileal content samples were dried overnight in a hot oven at 75°C and stored at -80°C. Samples then were sent to the Agricultural Experiment Station Chemical Laboratories (ESCL), University of Missouri, Columbia, MO, USA. Crude protein and crude fat concentrations were determined in the feed and the dried ileal digesta samples using standardized methods (Combustion Analysis (LECO) AOAC Official Method 990.03, 2006) and (Ether Extraction, AOAC Official Method 920.39 (a)), respectively [28]. The apparent ileal digestibility was calculated as proposed by Edwards and Gillis [29]:

$$Apparent\;ileal\;Digestibility\;(AID)=100–\left[100\times \frac{Ti\;in\;diet\;(ppm)}{Ti\;in\;ileum\;(ppm)}\times \frac{\%\;nutrient\;in\;ileum}{\%\;nutrient\;in\;diet}\right]$$

### Nucleic acid extraction

mRNA was extracted from the ileal tissue samples using the trizol-chloroform method. Precisely, 100 mg of frozen ileal tissue was homogenized with 1 mL of trizol using Benchmark BeadBlaster Microtube Homogenizer^®^ (OVERSTOCK Lab Equipment, NH, USA), followed by phase separation by adding 0.2 mL of chloroform, vortex mixing, incubation at room temperature (RT) for 3 minutes, and centrifugation (12000 rpm, 4°C, for 15 min). Carefully, 550μL of the aqueous phase was transferred into a tube containing 0.5 mL of isopropanol for RNA precipitation, vortexed and incubated at RT for 10 min. The tubes were then spun by centrifugation (12000 rpm, 10 min, 4°C), and the supernatant was removed. The pellet was washed with 1mL of 75% ethanol by vortexing, followed by centrifugation (12,000 rpm, 5 min, 4°C). After supernatant removal, the tube containing RNA pellet was incubated in RT for 10 min. The pellet was re-suspended in 100μL of nuclease-free water followed by incubation at 55–60°C for 10 min, then stored at -80°C. RNA was cleaned using the RNeasy Mini Kit (Qiagen, Hilden, Germany) according to the manufacturer`s protocol.

### Gene expression

The concentrations of the purified RNA samples were measured using a NanoDrop 2000 Spectrophotometer (Thermo Fischer Scientific, DE, USA) and then diluted to the concentration of 200 ng/μl by nuclease-free water. Reverse transcription was performed by adding 10 μL of diluted RNA to High-Capacity cDNA Reverse Transcription Kit (Thermo Fischer Scientific, MA, USA) according to the manufacturer’s protocol, using a Gradient Mastercycler (Eppendorf, NY, USA) adjusted for repeated cycles: 10 minutes at 25°C, 120 minutes at 37°C, 5 minutes at 85°C, and the final cycle at 4°C. Transcribed cDNA samples were stored at -25°C. The concentration of each samples’ cDNA was measured using NanoDrop 2000 Spectrophotometer and then was diluted to 20 ng/μl prior to quantitative analysis. Each RT-qPCR reaction was composed of 2 μL of diluted cDNA, 0.3 μL of each forward and reverse primers (10μM) (S2 Table), 7.4 μL of nuclease-free water, and 10 μL of Fast SYBR^TM^ Green Master Mix^®^ (Applied Biosystems, CA, USA). Each reaction was run in triplicates. The RT-qPCR was conducted using StepOnePlus (Applied Biosystems, CA, USA) and set to 50°C for 120s, then 95°C for 120s, proceeded by 40 cycles of 95°C for 15s and 60°C for 60s, with measuring C_t_ values at the endpoint of each cycle and recording the melting temperature curve. The threshold cycle values (C_t_) of interest genes were normalized against the C_t_ values of the β-actin gene (endogenous control), and the fold increase was calculated versus the control group (TNc). The relative mRNA expression was analyzed using 2^-ΔΔ*Ct*^ method [30] for the following genes; sugar transporters: SGLT1, GLUT1, GLUT2, GLUT5, GLUT8, GLUT10, and GLUT12; fatty acid transporters: FABP1, FABP2, FABP6, and FATP1; peptide transporters: PEPT1, PEPT2, and PHT1; villus enterocytes markers: ACSL5 and IAP; and oxidative genes: iNOS and CYBB.

### Histology and ileum morphological analysis

A 2 cm segment of ileum tissue was collected from each of seven randomly selected individuals from each treatment group and immediately fixed in 10% buffered formaldehyde solution. The fixed tissue samples were washed, alcohol dehydrated, trimmed into cassettes, and then embedded into paraffin. The tissue was then sectioned and stained with hematoxylin and eosin (H&E). The stained sections were examined, and images were captured using KEYENCE BZ-X810^®^ (Osaka, Japan) at 100x magnification for illustration. The ileum tissue images were captured at 40x magnification and then ImageJ software (NIH, Bethesda, MD) was used to acquire the following indices: The villus height from the villus tip to the companion crypt, the crypt depth from the crypt top to the submucosa, and the ratio of villus height to crypt depth (villus:crypt). All the intact straight villi were measured after which five villus-crypt measurements were randomly selected for each sample using an online randomizing website (Randomlists.com) prior to statistical analysis (n = 35 measurement per treatment).

### Statistical analysis

The statistical analysis was established with the SAS^®^ Studio software (SAS, 2018), using a two-way ANOVA model. The statistical model implemented was:

$$y_{ijk}=\mu +\alpha_{i}+\beta_{j}+({\alpha \beta )}_{ij}+\varepsilon_{ijk}.$$

where μ is the overall mean of the response, *α*_*i*_ is the effect of temperature, where i = 20 or 35; *β*_*j*_ is the effect of infection status, where j = infected or uninfected; *(αβ)*_*ij*_ represents the interaction of temperature and infection, and the *ε*_*ijk*_ is the random error. We analyzed the cloacal temperature, growth, feed intake, feed conversion ratio (FCR), apparent nutrients digestibility, the nutrients transporter expression, the mRNA expression of the absorptive enterocyte gene markers, oxidative genes, and ileum morphology parameters among the groups. Differences among treatments were tested using the “Tukey’s HSD” option. The significant differences between treatment groups (TNc, TNi, HSc, and HSi) were declared at *p*<0.05.

## Results

### Heat stress and *E*. *maxima* infection impact the chickens’ performance

**Fig 1:**
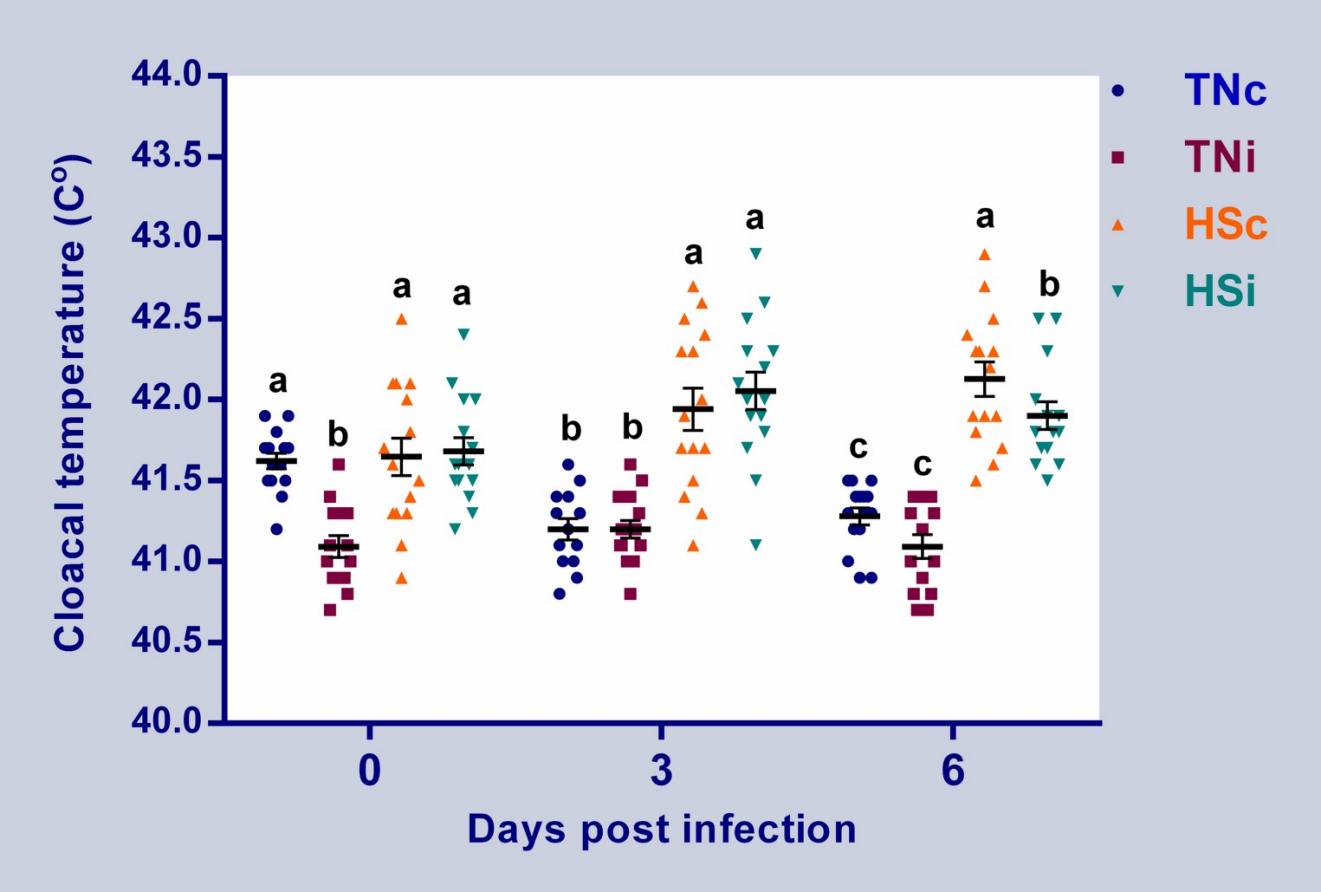
Cloacal temperature. Cloacal temperature of chickens infected with *E*. *maxima* and their uninfected controls that are raised in a thermoneutral or heat stress environment: TNc = thermoneutral control, TNi = thermoneutral infected, HSc = heat stress control, HSi = heat stress infected. Significant differences (p < 0.05) were depicted by different letters. The error bars represent the SEM.

**Fig 2:**
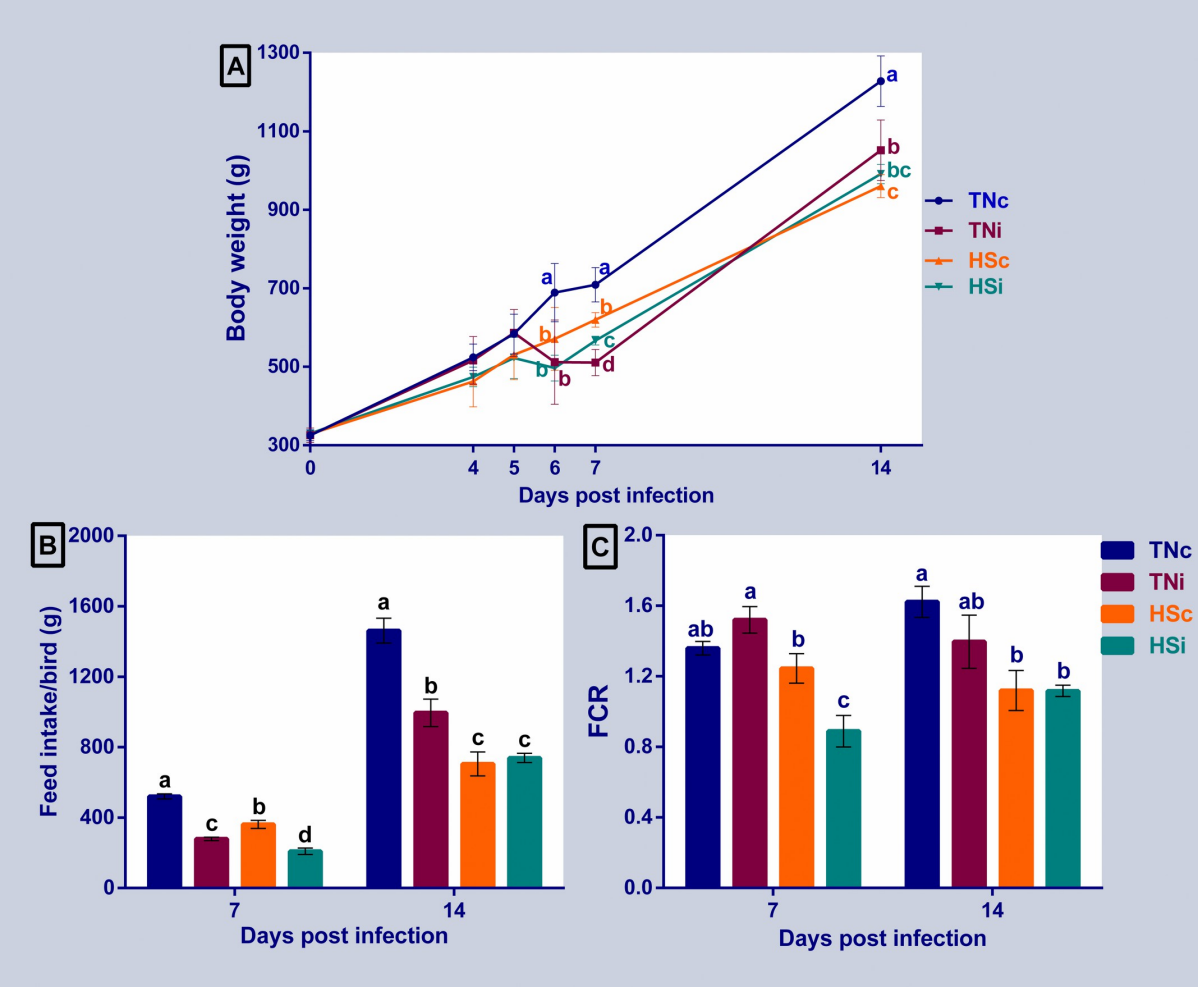
Chickens’ growth, feed intake, and FCR. The average body weight at 4, 5, 6, 7, and 14 dpi (A), feed intake at 7 and 14 dpi (B), and feed conversion ratio (FCR) at 7 and 14 dpi (C) of chickens infected with *E*. *maxima* and their uninfected controls that are raised in a thermoneutral or heat stress environment: TNc = thermoneutral control, TNi = thermoneutral infected, HSc = heat stress control, HSi = heat stress infected. Significant differences (p < 0.05) were depicted by different letters. The error bars represent the SEM.

The cloacal temperature records revealed a significant increase (0.6–1.0°C) in the HS groups (infected and control) at 3 and 6 dpi, compared with the TN groups (Fig 1). Both HS and *E*. *maxima* infection affected the growth of the chickens (Fig 2A). All the treatment groups showed a significant reduction in growth at 6 dpi (P<0.05), compared with the TNc group. The infected groups (HS and TN) had lower average body weight than the HSc group, but the difference was not significant. The TNi had the lowest average body weight at 7 dpi, followed by the HSi group and then the HSc group when compared to the TNc group, and all the differences in means were significant (P<0.05). At 14 dpi, the TNc group had the highest (P<0.05) body weight with no significant differences among the TNi and HSi or between HSc and HSi groups. The HSi group showed the lowest average body weight (Fig 2A). Heat stress and *E*. *maxima* infection also affected feed intake and FCR in chickens as illustrated in Fig 2B and 2C, respectively. The cumulative feed intake of the TNi, HSc, and HSi groups were significantly reduced by 47%, 31%, and 60% at 7 dpi, and 32%, 52%, and 51% at 14 dpi when compared to the TNc group, respectively (Fig 2B). At 7 dpi, the HSi had the lowest FCR compared to the other groups (*P*<0.05), and there were no differences among TNc and HSc (Fig 2C). At 14 dpi, the FCR of the TN groups were not significantly different but they were both higher than that in HS groups. However, the differences between the FCR of the TNi chickens and the HS chickens (control and infected) were approaching significance (p = 0.078 and 0.076, respectively).

### *E*. *maxima* infection reduces the nutrients digestibility

**Fig 3:**
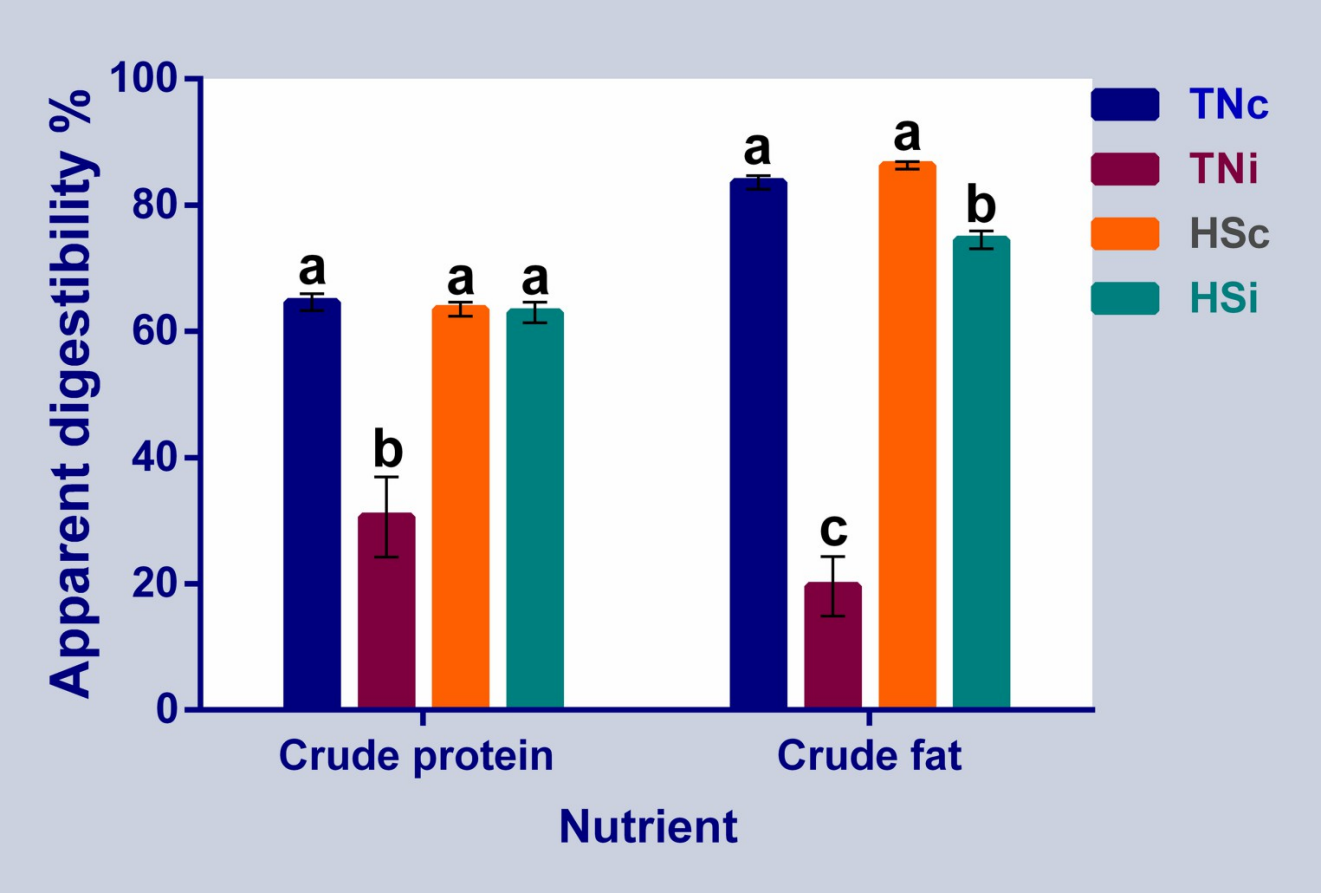
Chickens’ ileal crude protein and fat digestibility. The crude protein and crude fat apparent ileal digestibility at 6 dpi of chickens infected with *E*. *maxima* and their uninfected controls that are raised in a thermoneutral or heat stress environment: TNc = thermoneutral control, TNi = thermoneutral infected, HSc = heat stress control, HSi = heat stress infected. Significant differences (p < 0.05) were depicted by different letters. The error bars represent the SEM.

The AID for crude protein decreased by 50% in the TNi group compared to the other groups (Fig 3), and there were no differences among the TNc, HSc and HSi groups. The AID for crude fat was the lowest in the TNi group and decreased by 77% compared to the TNc group (Fig 3), and there was no difference in crude fat AID between the TNc and HSc; whereas, HSi had significantly lower (14%) crude fat AID compared to the TNc group (Fig 3).

### Both heat stress and *E*. *maxima* infection alters the mRNA expression of the ileum nutrient transporters

**Fig 4:**
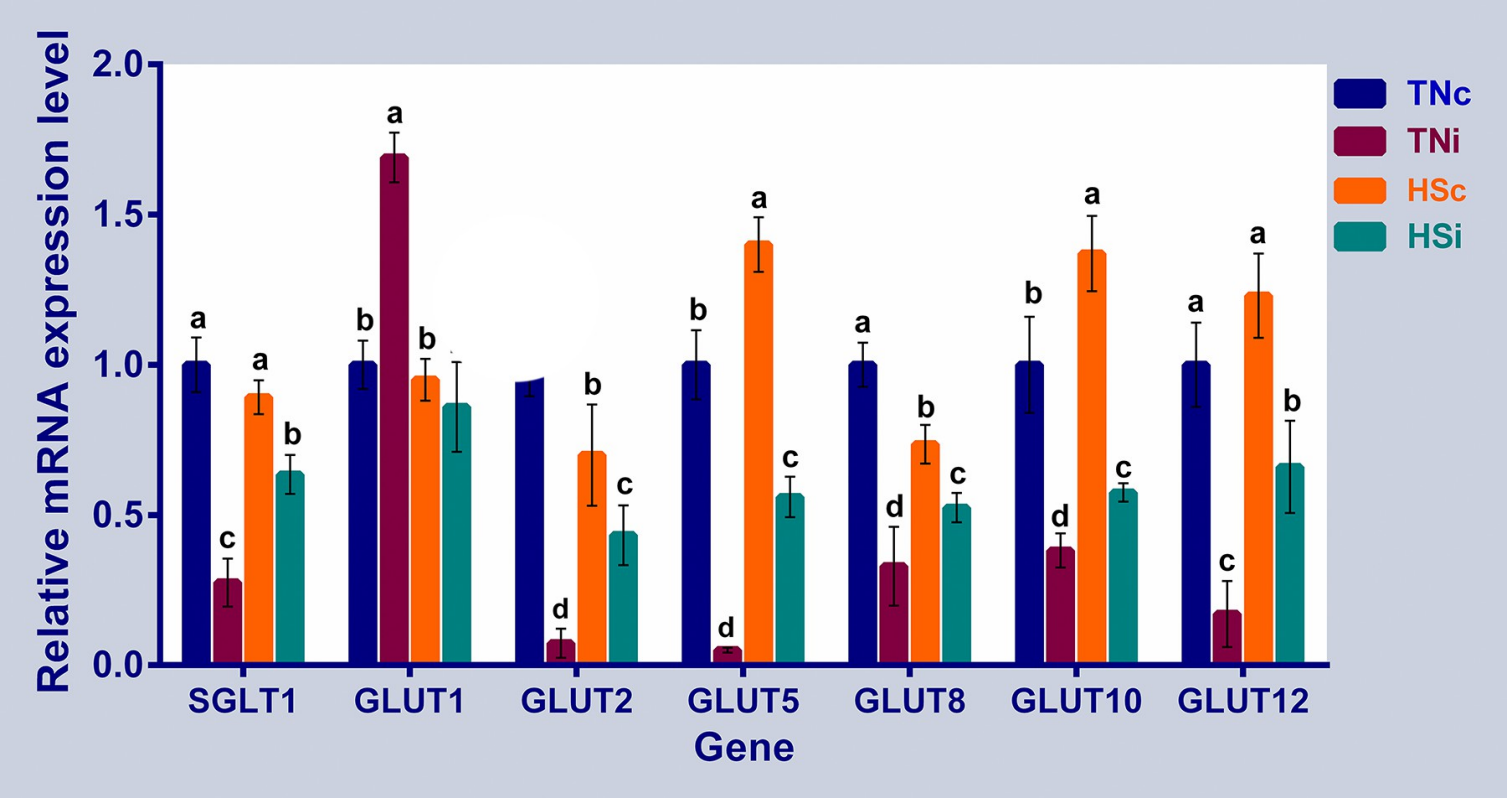
The mRNA expression of the ileal hexose transporters. The mRNA fold expression of the ileal hexose transporters at 6 dpi of chickens infected with *E*. *maxima* and their uninfected controls that are raised in a thermoneutral or heat stress environment: TNc = thermoneutral control, TNi = thermoneutral infected, HSc = heat stress control, HSi = heat stress infected. Significant differences (p < 0.05) were depicted by different letters. The error bars represent the SEM.

The mRNA expression of the ileal hexose transporters are depicted in Fig 4. SGLT1 and GLUT2 were downregulated in the infected groups (TN and HS) compared to the TNc group. GLUT1 was upregulated in the TNi group compared to the TNc group. The expression levels of GLUT5, 10, and 12 were downregulated in the infected groups but upregulated in the HSc group compared with the TNc group. GLUT8 expression was downregulated in all treatment groups compared to the TNc group. The HSi chickens’ expression levels of all investigated hexose transporters except GLUT1 were higher than those in the TNi group but lower than the expression levels in the HSc chickens.

**Fig 5:**
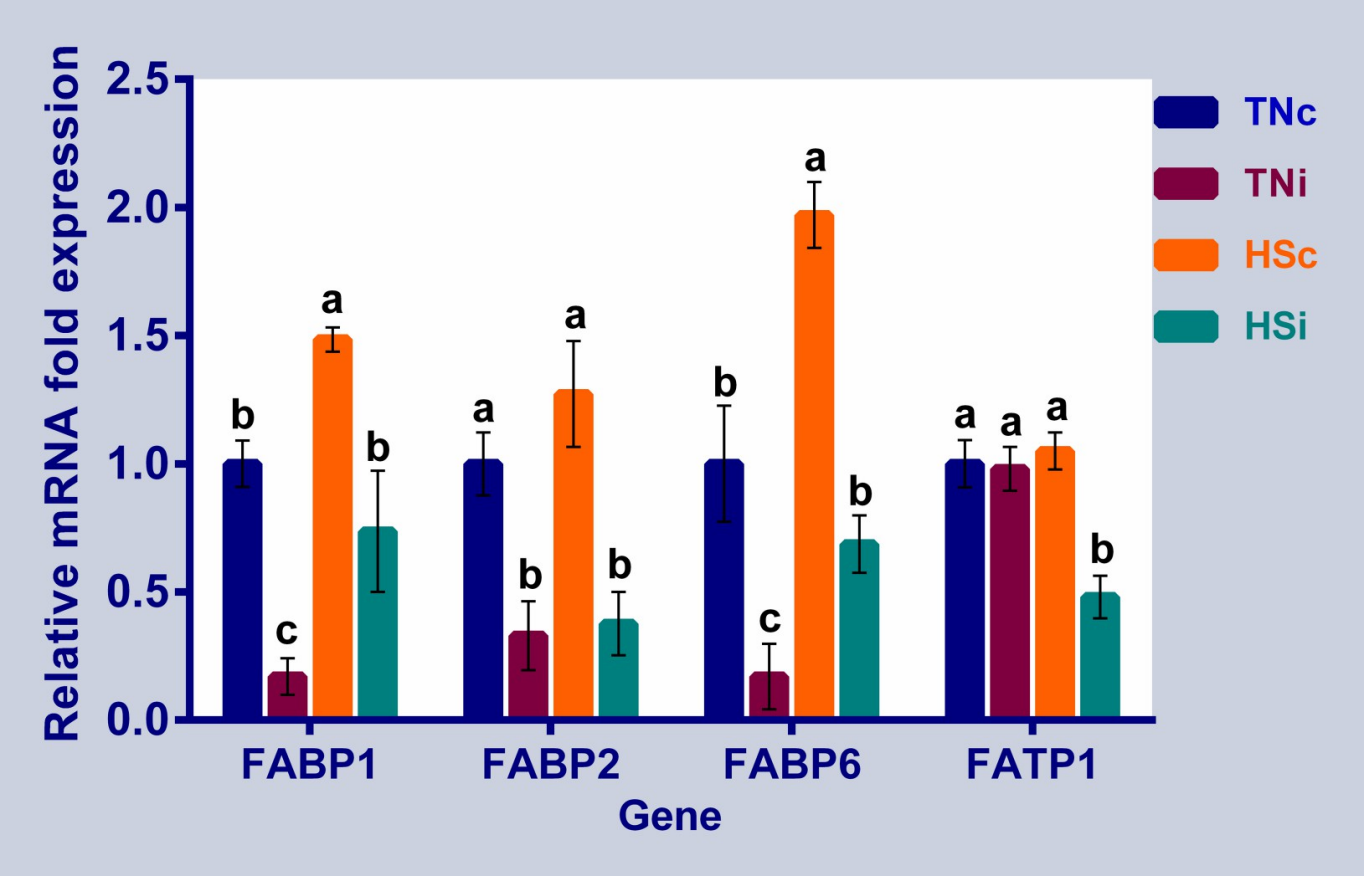
The mRNA expression of the ileal fatty acid transporters. The mRNA fold expression of the ileal fatty acid transporters at 6 dpi of chickens infected with *E*. *maxima* and their uninfected controls that are raised in a thermoneutral or heat stress environment: TNc = thermoneutral control, TNi = thermoneutral infected, HSc = heat stress control, HSi = heat stress infected. Significant differences (p < 0.05) were depicted by different letters. The error bars represent the SEM.

The expression levels of the ileal fatty acid transporters are displayed in Fig 5. The mRNA expression of FABP1 and FABP6 were downregulated in the TNi group but upregulated in the HSc group compared to the TNc and TNi groups. FABP2 was downregulated in the infected groups compared to uninfected groups. The expression levels of FABP1 and 2 were higher in the HSi group than the TNi group. Only HSi group exhibited downregulation of the FATP1.

**Fig 6:**
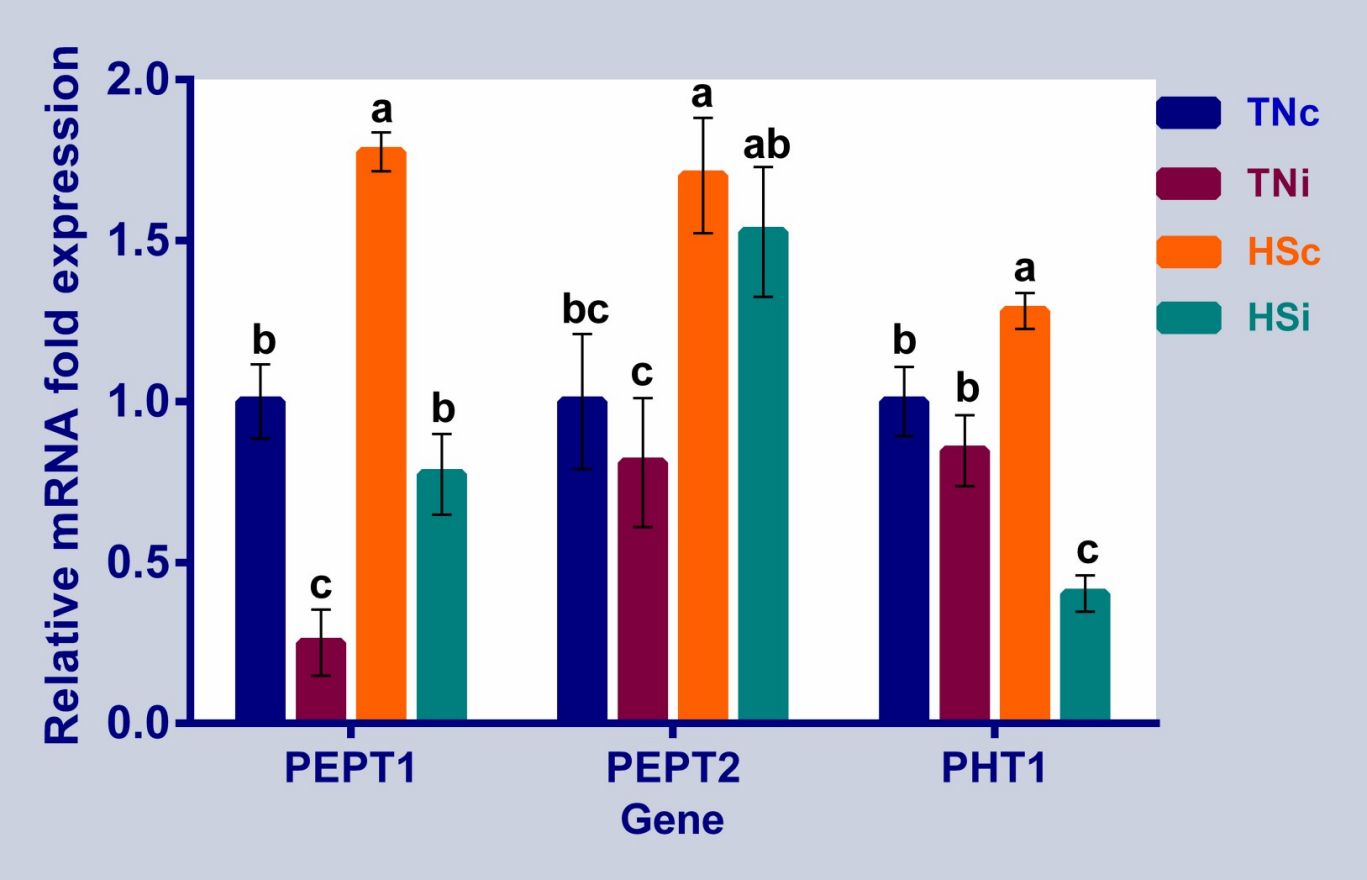
The mRNA expression of the ileal peptide transporters. The mRNA fold expression of the ileal peptide transporters at 6 dpi of chickens infected with *E*. *maxima* and their uninfected controls that are raised in a thermoneutral or heat stress environment: TNc = thermoneutral control, TNi = thermoneutral infected, HSc = heat stress control, HSi = heat stress infected. Significant differences (p < 0.05) were depicted by different letters. The error bars represent the SEM.

The mRNA expression of the ileal peptide transporters is presented in Fig 6. The expression of PEPT1 was downregulated in the TNi group and upregulated in the HSc group compared to the TNc and TNi groups. PEPT2 was upregulated in the HSc group compared to the TN groups (control and infected). The HSi group showed a significant higher expression level of PEPT2 compared with the TNi group. mRNA expression of PHT1 was upregulated in the HSc group but downregulated in the HSi group, when compared with the TN groups. Interestingly, the mRNA expression levels of SGLT1, GLUT2, GLUT5, GLUT8, GLUT10, GLUT12, FABP1, FABP6, PEPT1, and PEPT2 in the HSi chickens were significantly higher than those in the TNi chickens.

### The effect of HS and *E*. *maxima* infection on the ileum epithelial lining

**Fig 7:**
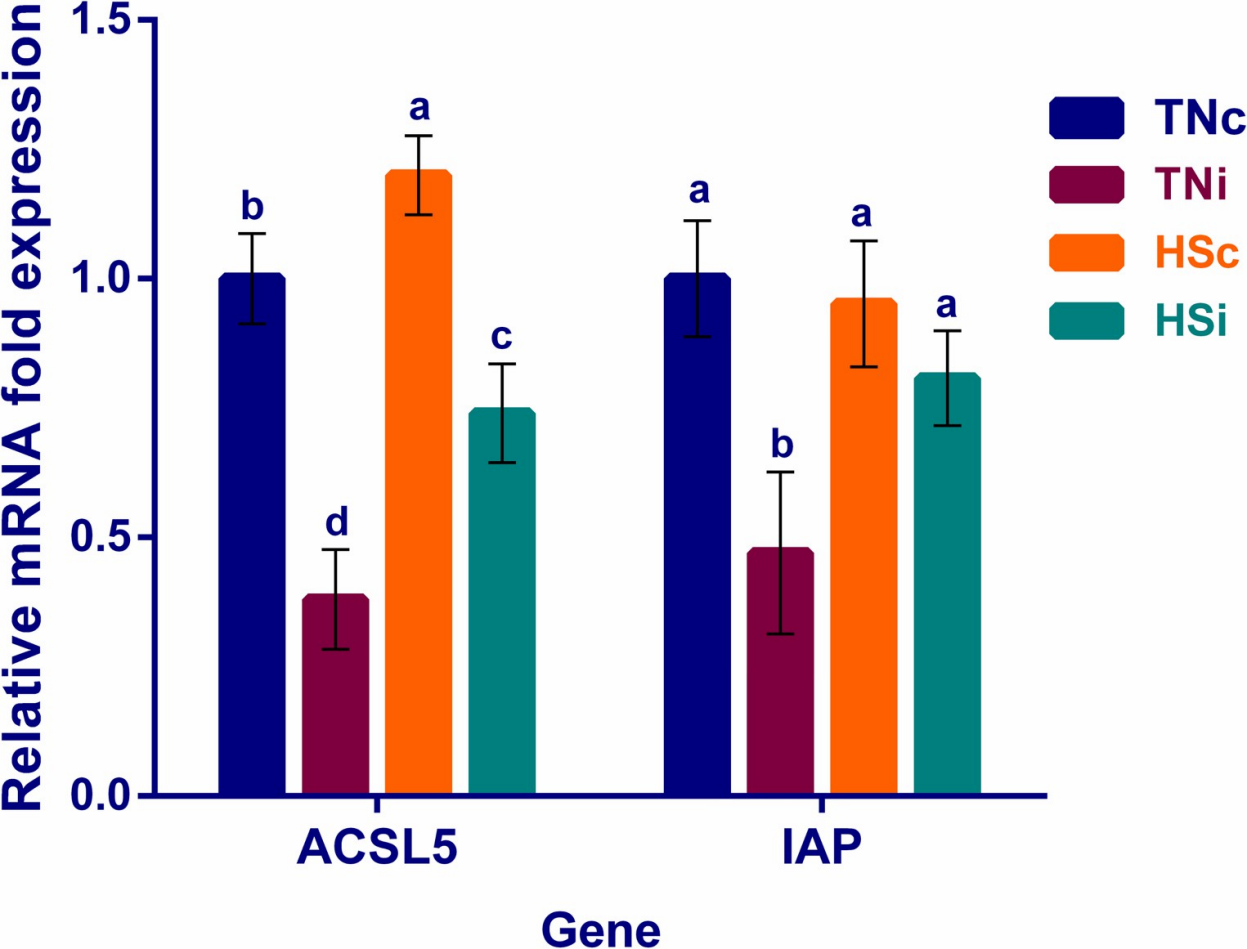
The mRNA expression of the ileal villus absorptive enterocytes’ marker genes. The mRNA fold expression of marker genes specific for ileal villus absorptive enterocytes at 6 dpi of chickens infected with *E*. *maxima* and their uninfected controls that are raised in a thermoneutral or heat stress environment: TNc = thermoneutral control, TNi = thermoneutral infected, HSc = heat stress control, HSi = heat stress infected. Significant differences (p < 0.05) were depicted by different letters. The error bars represent the SEM.

The TNi group had the lowest mRNA expression levels of both ACSL5 and IAP, compared with the other treatment groups as shown in Fig 7. The ACSL5 was upregulated in the HSc group and downregulated in the HSi group, compared with the TNc group.

**Fig 8:**
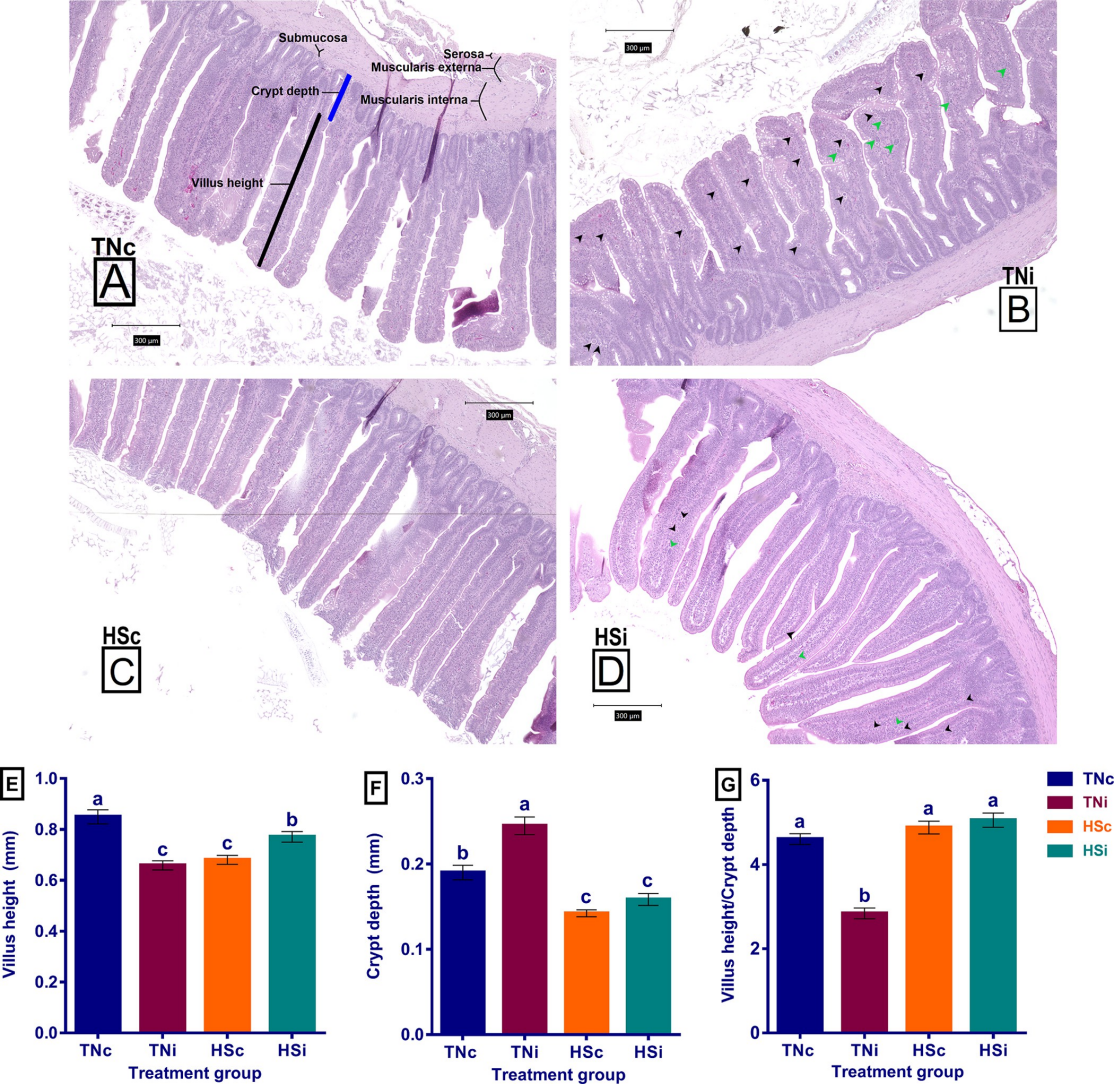
The ileal histopathology and morphological parameters. The histopathology picture: Photomicrograph of ileum tissue stained with hematoxylin and eosin (H&E), at 100x magnification, of TNc (A), TNi (B), HSc (C), and HSi (D); the villus height (E); the crypt depth (F); the villus height to crypt depth ratio (G); at 6 dpi of chickens infected with *E*. *maxima* and their uninfected controls that are raised in thermoneutral or heat stress environment: TNc = thermoneutral control, TNi = thermoneutral infected, HSc = heat stress control, HSi = heat stress infected. Significant differences (p < 0.05) were depicted by different letters. The error bars represent the SEM. The ileum histological components were illustrated (A). The arrow heads point the *E*. *maxima* stages (B, D) gametocytes (black arrow heads) and schizonts (green arrowheads).

The morphology of the ileum tissue is displayed in Fig 8A–8D showing marked crypt depth increase and villus swelling, shortening, and irregularity in the TNi group, compared with the other treatment groups. A significant reduction in villus height was observed in the TNi, HSc, and HSi groups, compared with the TNc group, as demonstrated in Fig 8E. However, the HSi group showed higher villus average than the TNi and HSc groups (p = 0.016). The crypt depth is illustrated in Fig 8F. The TNi group had the deepest crypts, compared with the other treatment groups. Both HS groups (infected and control) expressed a decrease in crypt depth, compared with the TNc group. The villus:crypt ratio is displayed in Fig 8G, revealing that the TNi group had the lowest ratio.

**Fig 9:**
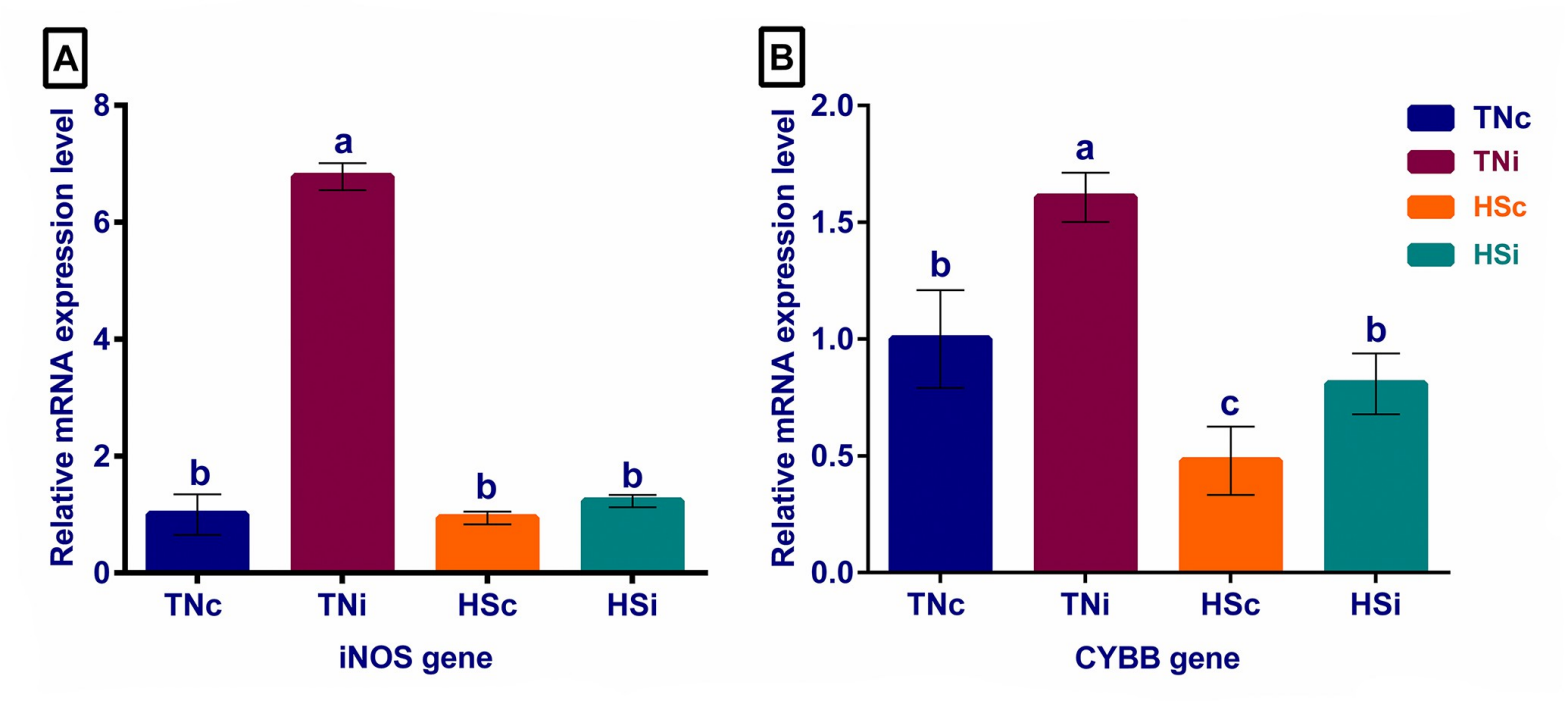
The mRNA expression of ileum tissue oxidative gene. The mRNA fold expression of ileum tissue oxidative genes iNOS (A) and CYBB (B) at 6 dpi of chickens infected with *E*. *maxima* and their uninfected controls that are raised in a thermoneutral or heat stress environment: TNc = thermoneutral control, TNi = thermoneutral infected, HSc = heat stress control, HSi = heat stress infected. Significant differences (p < 0.05) were depicted by different letters. The error bars represent the SEM.

The expression levels of the oxidative enzymes are displayed in Fig 9. The TNi group showed a 7-fold increase in iNOS expression and at the least 1.6-fold increase in CYBB expression, compared with the other treatment groups. The CYBB expression was significantly downregulated in the HSc group, compared with the TNi and HSi groups.

## Discussion

### *Eimeria maxima* infection and HS affects growth and feed intake

Meat-type (broiler) chickens consume a large amount of feed thereby increasing the internal heat load produced by digestion and metabolism. Under HS, broilers reduce the feed intake to minimize internal heat production. In the current study, exposing chickens to HS significantly increased cloacal temperature indicating the effectiveness of heat treatment (Fig 1) [31, 32]. Expectedly, the growth and feed intake were markedly impacted by HS, *E*. *maxima*, and their combination. The largest growth depressions were observed in the infected groups at 6 and 7 dpi. However, at 14 dpi, the HS groups suffered the largest growth depression. Interestingly, the HSi group showed faster growth recovery from *E*. *maxima* impact than the TNi group suggesting that the *E*. *maxima* effect on growth may be transient while the HS has the most persistent impact. The HSi group also showed better feed conversion than the TNi group (Fig 2C). Taken together, the data from the current study suggests that, even though the effect of HS on performance of broiler chickens may linger longer than that of *E*. *maxima* infection, HS may also curtail the adverse effect of *E*. *maxima* infection on performance.

### *Eimeria maxima* infection and heat stress affect nutrient digestibility

*Eimeria* parasites invade and destroy the intestinal epithelium causing a severe reduction in nutrient digestibility [20, 22]. On the other hand, HS was found to induce intestinal leakage [32], Therefore, it was expected that the combined effects of HS and *E*. *maxima* infection will significantly destroy the intestinal epithelium and impair digestibility. In the current study, the AID for crude protein and crude fat were reduced in the TNi group. However, it seems that the infected birds that were reared under HS had a slight reduction in AID for crude fat and no reduction in AID of crude protein (Fig 3). The expected negative effects of *E*. *maxima* on AID were modulated by the HS. Schneiders *et al*. [33] showed that HS limits the reproductive ability of *E*. *maxima*. The heat treatment may have interfered with the development of *E*. *maxima* thereby limiting its ability to affect ileum digestibility.

### *Eimeria maxima* infection and heat stress affect nutrient transporters

#### Hexose transporters

The absorption of simple sugars in the intestine is essential for energy balance and body glucose homeostasis [34]. Monosaccharides are transported through a group of active and passive transports. SGLT1 is an active Na^+^/glucose symporter that is mainly expressed on the enterocytic apical membrane and utilizes the concentration gradient of the Na^+^ to transport the glucose against its concentration whereas GLUT1, 2, 5, 8, 10, and 12 are facilitative sugar transporters that allow the movement of sugars along their concentration gradient and have different sugar-binding affinity and insulin sensitivity [12, 14]. The expression of both SGLT1 and GLUT2 transporters was significantly reduced in the infected groups, compared with the TNc group (Fig 4). Similar results were observed in the enterocytes of nematode infected mice in which the activated M2 macrophages inhibited the SGLT1 activity without downregulation. The SGLT1 inactivation, accompanied by the GLUT2 downregulation, resulted in compensatory upregulation of GLUT1 as suggested by Notari *et al*. [35]. Similarly, we observed an upregulation of GLUT1 in the TNi group compared with other treatment groups which is in concordance with the report by Notari *et al*. [35] and Teng *et al*. [36]. Furthermore, SGLT1 inactivation and GLUT2 downregulation were also observed in the enterocytes of rats exposed to different physical stressors [37, 38], and in the jejunum of broiler chicken exposed to HS [39]. The immigrated macrophages and lymphocytes into *E*. *maxima* infected sites are mainly expressing GLUT1 that may also contribute to the observed upregulation of GLUT1 in the ileal tissue of the TNi birds, compared with the other treatment groups (Fig 4) [40, 41].

The downregulation of GLUT2 and GLUT5 transporters in the TNi group is in concordance with studies on *E*. *maxima* infected broiler chicken [42–44]. Heat stress was found to increase insulin secretion in humans [45], rats [46], ruminants [47], and chickens [48, 49]. Thus, the upregulation of the insulin-sensitive GLUT10 and GLUT12 transporters in the HSc group compared to the TNc group may be under the influence of insulin elevation [12]. Contrarily, the mRNA expression levels of the GLUT1, GLUT10, and GLUT12 were downregulated in the ileal tissue of heat-stressed laying hens [50]. The differences in these results could be due to the genetic differences between layers and meat-type chickens.

GLUT8 was downregulated in the infection groups relative to their uninfected controls. GLUT8 regulates enterocytic fructose transport in a negative correlation manner by modulating the abundance and membrane utilization of GLUT12 [51]. Coincidentally, GLUT8 was downregulated while GLUT12 and GLUT5 were upregulated in the HSc group possibly to meet the energy demand. The independent expressions of SGLT1 and GLUT5 under HS were similar to the report of Habashy *et al*. (2017). In the current study, we show that HS modulates the mRNA expression of hexose transporters in the ileum of broiler chickens infected with *E*. *maxima*.

#### Fatty acid transporters

The majority of dietary lipids are absorbed along the intestine through a group of fatty acid transporters and binding proteins [52]. Therefore, the enterocyte contains a FABP-rich cytosol: the liver-type FABP (FABP1) was first identified in the liver and later on was found to be abundantly expressed in the intestine, the intestinal-type FABP (FABP2) was found exclusively present in the enterocytes [53], and the ileal lipid or bile-acid binding protein (FABP6) has a higher affinity to bind bile acids than lipids [54]. The HSc group showed upregulation of FABP1 that was similarly reported in response to immune stress [55], chronic heat stress [11], and corticosteroid administration [56] in broiler chickens, as well as rats under corticosteroid treatment [57]. The role of cortisol in upregulating the expression of FATP1 was possibly due to the interaction of FATP1 with the cytoplasmic steroid hormone receptor HNF4α that acts as a transcriptional factor regulating different cellular functions, including the genes associated with inflammation and oxidative stress in liver and intestine [58–60]. We can hypothesize that the upregulation of FABP1 under HS is essential to mitigate the oxidative stress effect through regulating lipid signaling and phospholipid incorporation. It was found that the incorporation of phospholipids into cells was increased by the presence of FABP1 [61–63]. The observed upregulation of the FABP6 expression in the HS groups (control and infected) compared with TN groups (control and infected), respectively, could be explained by the enterocytes increasing transportation capacity to reabsorb the bile acids over-secretion induced by plasma hydrocortisone level [64–66]. Regarding the mucosal injury caused by the *E*. *maxima* infection, the downregulation of FABPs was a plausible outcome (Fig 5). However, the expression levels of FABP1 and FABP6 in the HSi group were significantly higher than the TNi group suggesting that ileum epithelial damage in the TNi was substantial compared to that of the HSi group. Unlike FABPs, FATP1 is only expressed on the membrane and not in the cytoplasm. FATP1 is the primary candidate for absorbing long-chain fatty acids (LCFA) and is believed to facilitate the bidirectional movement of LCFA across the plasma membrane [67]. Since the HSi group had the lowest feed intake 7 dpi (Fig 5), it is possible that the enterocyte downregulated the FATP1 expression to the level only required for absorption.

#### Peptide transporters

Digestible protein is broken down and hydrolyzed along the gut releasing small peptides and free amino acids that are absorbed through the intestinal mucosa [68]. Oligopeptide transporters of the SLC15A1 family, including PEPT1, PEPT2, and PHT1, are responsible for proton-coupled active transporting of di- and tripeptides and expressed on the apical membrane of the enterocytes as well as various types of body cells [19, 69]. The upregulation of PEPT1 and PEPT2 expression in HS groups (infected and control) compared with the TN groups (infected and control), respectively (Fig 6), may be under glucocorticoid influence through the stress-related serum and glucocorticoid-regulated kinase 1 (SKG1) [70–72]. Similarly, PEPT1 upregulation was observed in the small intestine of the corticosteroid-treated broiler chicken [56]. In other studies, the jejunum expression of PEPT1 was downregulated in broiler chickens challenged with mixed *Eimeria* spp (*acervulina*, *praecox*, *maxima*, *mitis*) [73], and under experimentally developed necrotic enteritis [74]. While the PEPT1 was upregulated in the broiler ileal tissue, PEPT2 was downregulated after 12 days of continuous heat stress [11]. The PHT1 transporter is abundantly expressed on the endosomal and lysosomal membranes as well as the enterocytic membrane [75–77]. PHT1 was downregulated in the HSi group compared to the HSc group (Fig 6), maybe due to the combination of *E*. *maxima* with HS reduced the endosome abundance or function [78]. The cellular autophagy and endosome/lysosome responses under physical and infectious stresses require further investigation.

### Heat stress limits the *E*. *maxima*-induced villus enterocytic damage

Since the AID and mRNA expression of most ileal nutrient transporters were significantly improved in the HSi group compared with the TNi group, we suggest that HS may limit *E*. *maxima*’s ability to cause enterocytic damage. This hypothesis was further investigated using the expression levels of ACSL5, IAP, and SGLT1 that are considered molecular markers for the villus absorptive enterocytes [26, 79]. The SGLT1, ACSL5, and IAP expression levels were significantly higher in the HSi group than the TNi group (Figs 4 and 7). This suggests that either HS boosts the enterocytic function and gene expression and/or prevents the *E*. *maxima*-induced enterocytic destruction. The histopathology of the ileum tissues was examined to identify the ileal tissue damage in the four treatment groups (Fig 8). The intestinal morphology undergoes architectural changes including villi shortening and crypt deepening under conditions such as toxins, infection, and heat stress [80–82]. Similarly, the histological examination showed villus height reduction in all treatment groups (TNi, HSc, and HSi), compared with the TNc group. However, only the TNi group showed crypt hyperplasia, compared with TNc group (Fig 8A–8D). Interestingly, the TNi group showed a significantly shorter villus, deeper crypts, and smaller villus:crypt ratio than the HSi group suggesting that HS limited the ileum tissue damage caused by *E*. *maxima* (Fig 8E–8G).

### Ileum expression levels of oxidative genes

The two major causes of the *Eimeria*-induced villus enterocytic damage are the parasite invasion and replication, and the oxidative radicals produced from macrophages and heterophils during the immune response. It has been reported that HS curtails the *E*. *maxima* life cycle and inhibits its gametogony [33]. The ileum tissue expression of the oxidative genes iNOS and CYBB were assessed to investigate the other cause of the enterocytic damage. Activated chicken macrophages and heterophils express iNOS and CYBB genes that are responsible for nitric oxide and hydrogen peroxide production, respectively [83–85]. The significant upregulation of both iNOS and CYBB genes in the TNi group, compared with the HSi group, suggests a higher tissue production of oxidative radicals (•NO and H_2_O_2_). It is therefore plausible that reactive oxygen species contributed to the tissue deterioration detected mostly in the TNi group. We expected HS to exacerbate the effects of *E*. *maxima* infections in broiler chickens, however, the results of the current study strongly suggest that HS rather curtailed the negative impact of *E*. *maxima* on ileum function and tissue morphology by putatively suppressing the oxidative damage of the enterocytes.

## Conclusion

The current study demonstrates that HS and *E*. *maxima* infection, either individually or combined, impact growth and feed intake of broiler chickens. Even though *E*. *maxima* and HS are independent stressors in broiler chickens, their combined effects are not synergistic. It appears that HS curtailed the effects of the *E*. *maxima* infection resulting in the outcomes of apparent digestibility and mRNA expression of nutrient transporters of the infected chickens reared under heat stress to be similar to their uninfected counterparts reared under heat stress. Exposing the *E*. *maxima* infected broiler chickens to HS curtailed the ileum tissue injury and suppressed the immune-induced oxidative damage to the enterocytes. Further molecular mechanistic studies are recommended to identify the key cellular and molecular events associated with the HS effect on the *E*. *maxima* life cycle inside the broiler chicken’s intestine.

## Supporting information

S1 Table The analysis of the feed. (PDF)

S2 Table Primer pairs used for RT-qPCR analyze of the ileum genes’ expression levels. (PDF)
