# Supplementary material for: Heat stress modulates the disruptive effects of Eimeria maxima infection on the ileum nutrient digestibility, molecular transporters, and tissue morphology in meat-type chickens
Source: PLoS One. 2022 Jun 3;17(6):e0269131. doi: 10.1371/journal.pone.0269131 (PMC9165794; doi:10.1371/journal.pone.0269131)
Supplement: S1 Table — (PDF) [file pone.0269131.s001.pdf]

Supplementary table 1 (S1): The analysis of the feed

Element	%
Taurine	0.19
Hydroxyproline	0.03
Aspartic acid	2.05
Threonine	0.78
Serine	0.88
Glutamic acid	3.57
Proline	1.09
Lanthionine	0.02
Glycine	0.84
Alanine	0.99
Cysteine	0.32
Valine	1.00
Methionine	0.43
Isoleucine	0.91
Leucine	1.68
Tyrosine	0.64
Phenylalanine	1.02
Hydroxylysine	0.03
Ornithine	0.02
Lysine	1.16
Histidine	0.54
Arginine	1.32
Tryptophan	0.26
Gross energy (Kcal/100 g)	362.80
Crude protein	18.76
Moisture	11.02
Crude fat	5.64
Crude fiber	2.63
Ash	5.33
