# Supplementary material for: Heat stress modulates the disruptive effects of Eimeria maxima infection on the ileum nutrient digestibility, molecular transporters, and tissue morphology in meat-type chickens
Source: PLoS One. 2022 Jun 3;17(6):e0269131. doi: 10.1371/journal.pone.0269131 (PMC9165794; doi:10.1371/journal.pone.0269131)
Supplement: S2 Table — (PDF) [file pone.0269131.s002.pdf]

Supplementary table 2 (S2): Primer pairs used for RT-qPCR analyze of the ileum genes' expression levels

Description	Gene	Gene bank Accession Number	Size	Align.	Primers Sequences
Glucose transporters	SGLT1 (SLC5A1)	NM_001293240	97	Forward	5′GAGGAGAAACCCGATGAAAGAG3′
				Reverse	5′CTAAGCCACAGAACCAGTTGTA3′
	GLUT1 (SLC2A1)	NM_205209.1	105	Forward	5′CTTCTGCATACACTCCTTCTCC3′
				Reverse	5′TGGACGTGAAACCAGCTAAA3′
	GLUT8 (SLC2A8)	AB083371	309	Forward	5′GCAGCAGAGGTTATTCGCGCC3′
				Reverse	5′GCCTCCCAGTATTCCTCCAGC3′
	GLUT10 (SLC2A10)	XM_417383.5	133	Forward	5′CCGCTGCAGATGAGGTATTT3′
				Reverse	5′GTTTCTTCTCAGAGCCGTAGTG3′
	GLUT12 (SLC2A12)	XM_419733.5	110	Forward	5′AGAGAGTGGGGAGGTTCCC3′
				Reverse	5′TCAGGACGAGCCAAGACA3′
Fructose transporters	GLUT2 (SLC2A2)	NM_207178.1	577	Forward	5′ATGCTGGTGGTCAATGTCCTCTC3′
				Reverse	5′TGATGCCTGAGAACTGCTGCGAT3′
	GLUT5 (SLC2A5)	XM_417596.6	108	Forward	5′AGGCTGATCTCTGCCTTTG3′
				Reverse	5′GTCGATGTAGGTTCGGTTGTAG3′
Fatty Acids transporters	FABP1	NM_204192.3	94	Forward	5′CCAGAAGGGTAAGGACATCAAG3′
				Reverse	5′GGTCATTACTTTGGAGCCAGTA3′
	FABP2	NM_001007923.1	77	Forward	5′AAAGATAATGGAAAAGTACTCACAGCAT3′
				Reverse	5′CCTTCGTACACGTAGGTCTGTATGA3′
	FABP6	XM_015293653.2	59	Forward	5′CGGTCTCCCTGCTGACAAGA3′
				Reverse	5′CCACCTCGGTGACTATTTTGC3′
	FATP1 (SLC27A1)	NM_001039602.2	119	Forward	5′TGCCTTTACGCCAGGATTT3′
				Reverse	5′GAGGGTCTCACGTTGCTTATC3′

Peptide transporters	PepT1 (SLC15A1)	KF366603.1	105	Forward	5′TGGGAGTGAGAGTAGAAGTGAA3′
				Reverse	5′TCAGACCGCAAGCTAGAAAC3′
	PepT2 (SLC15A1)	KF366604.1	133	Forward	5′GTGGTCGGCCATCTGATAAA3′
				Reverse	5′CAGAGACACAGGGCTTGATAC3′
	PHT1 (SLC15A4)	XM_415099.6	62	Forward	5′AAGGCCAGGGAGTCCTTCA3′
				Reverse	5′TGACAGCTTAGCCATCTCAAACA3′
Absorptive villus' enterocytes gene-markers	ACSL5	NM_001031237.1	170	Forward	5′CCCTAAAGGTGCCATGCTGA3′
				Reverse	5′CTCCGCAGCTGTACATCACA3′
	IAP	XM_015291489.2	104	Forward	5′GAGCCTACACCAGCATCCTC3′
				Reverse	5′GCTGCCTGTAGTCCTTGTCC3′
Oxidative genes	iNOS	NM_204961.1	97	Forward	5′CAACAGGAACCTACCATCTGAC3′
				Reverse	5′GACCACTGGATTCTCCAAATAC3′
	CYBB	NM_001100286.1	113	Forward	5`AGCGAGGATGTGTTCAGTTATG3`
				Reverse	5`GTTGCATCGTGGCAGTATTTG3`
B-actin		NM 205518.1 125	125	Forward	5′AGACATCAGGGTGTGATGGTTGGT3′
				Reverse	5′TCCCAGTTGGTGACAATACCGTGT3′
